# Validation of the Italian Version of the Dizziness Handicap Inventory, the Situational Vertigo Questionnaire, and the Activity-Specific Balance Confidence Scale for Peripheral and Central Vestibular Symptoms

**DOI:** 10.3389/fneur.2017.00528

**Published:** 2017-10-10

**Authors:** Silvia Colnaghi, Cristiana Rezzani, Marco Gnesi, Marco Manfrin, Silvia Quaglieri, Daniele Nuti, Marco Mandalà, Maria Cristina Monti, Maurizio Versino

**Affiliations:** ^1^Department of Public Health, Experimental and Forensic Medicine, University of Pavia, Pavia, Italy; ^2^Laboratory of Neuro-otology and Neuro-ophthalmology, C. Mondino National Neurological Institute, Pavia, Italy; ^3^ENT Unit, Policlinico San Matteo Fondazione (IRCCS), Pavia, Italy; ^4^Department of Clinical-Surgical, Diagnostic and Paediatric Sciences, University of Pavia, Pavia, Italy; ^5^Department of Otology and Skull Base, Azienda Ospedaliera Universitaria Senese, Siena, Italy; ^6^Department of Brain and Behavioral Sciences, University of Pavia, Pavia, Italy

**Keywords:** Dizziness Handicap Inventory, Situational Vertigo Questionnaire, Activity-specific Balance Confidence scale, vertigo, vestibular, questionnaires

## Abstract

Neurophysiological measurements of the vestibular function for diagnosis and follow-up evaluations provide an objective assessment, which, unfortunately, does not necessarily correlate with the patients’ self-feeling. The literature provides many questionnaires to assess the outcome of rehabilitation programs for disequilibrium, but only for the Dizziness Handicap Inventory (DHI) is an Italian translation available, validated on a small group of patients suffering from a peripheral acute vertigo. We translated and validated the reliability and validity of the DHI, the Situational Vertigo Questionnaire (SVQ), and the Activities-Specific Balance Confidence Scale (ABC) in 316 Italian patients complaining of dizziness due either to a peripheral or to a central vestibular deficit, or in whom vestibular signs were undetectable by means of instrumental testing or clinical evaluation. Cronbach’s coefficient alpha, the homogeneity index, and test–retest reproducibility, confirmed reliability of the Italian version of the three questionnaires. Validity was confirmed by correlation test between questionnaire scores. Correlations with clinical variables suggested that they can be used as a complementary tool for the assessment of vestibular symptoms. In conclusion, the Italian versions of DHI, SVQ, and ABC are reliable and valid questionnaires for assessing the impact of dizziness on the quality of life of Italian patients with peripheral or central vestibular deficit.

## Introduction

The vestibular system may be affected by various pathologies causing disabling symptoms such as vertigo, nausea, postural instability, and falls. The life-long prevalence of dizziness in the general population is about 30%, the vestibular system function worsens with aging (1), and one subject out of three aged 65 or more experiences at least one fall per year (2). The diagnosis of the common vestibular pathologies as benign paroxysmal positional vertigo (BPPV), vestibular migraine (3), vertigo of central origin, vestibular neuritis or Meniere’s disease, is relatively simple during the acute phase based on the anamnesis and the clinical signs. Yet, after the acute phase or in the chronic patient, vestibular assessment becomes more difficult as the central nervous system activates very efficient adaptive and compensation mechanisms (4–9). Thus, a stabilized vestibular deficit can correspond either to a completely compensated condition, allowing the subject to perform even demanding tasks, or to a serious impairment, which may in turn improve with rehabilitation and still not change the neurophysiologic measurement. Actually, recovery of the vestibular function in patients with both peripheral and central vestibular disorders may be effectively stimulated using specific rehabilitation protocols (10–19). Outcome measures of vestibular rehabilitation are provided by questionnaires on the impact of disability in everyday life (20, 21). Several questionnaires are designed to measure these aspects, addressing different functional domains, and the aim of this study was to validate the use of three complementary instruments for the assessment of Italian patients with disequilibrium.

The original version of the Dizziness Handicap Inventory (DHI) is a 25-item questionnaire that asks the patients to rate their self-perception of disability from dizziness (20) and is the only one for which an Italian translation has been validated, but this was tested on a small group of patients, all suffering from peripheral vertigo and all evaluated in the acute phase of the disease (22). DHI consists of a 7-item physical subscale, a 9-item emotional subscale, and a 9-item functional subscale. A score of 4 points is assigned to a “yes” response, 2 points to “sometimes,” and 0 points to “no” response. Thus, the total score ranges from 0 (no perceived disability) to 100 (maximum perceived disability). The developers of the DHI ask to fill the questionnaire to 106 consecutive patients examined for vestibular testing, in whom, the mean score and SD was 32.7 ± 21.9. Scores of 60 or higher are associated with a greater fall risk in subjects with vestibular disorders (23) and the DHI score is sensitive to the effects of a 6- to 8-week course of vestibular rehabilitation in patients with unilateral, bilateral, central, or non-specific vestibular dysfunction (24).

The Activities-specific Balance Confidence scale (25) is a 16-item questionnaire designed for evaluating the risk of falls in everyday activities. The patient is asked to rate his perceived confidence in performing 16 activities that can take place inside and outside the house. Each activity can be scored from 0 (no confidence) to 100 (100% confident). The ABC scale and the DHI have been shown to have a moderately negative correlation, indicating that the ABC scale is a valid tool that can be administered to persons with vestibular dysfunction (26, 27). Older adults should be able to perform most of the 16 ABC activities with great confidence. Subjects who score less than 80% on the ABC scale are somewhat impaired (28), those who score less than 50% are often home-bound individuals, and older adults who score 66% or less are at high risk for falling (27). Furthermore, the ABC scale has also been shown to be sensitive to changes over the course of rehabilitation (23).

The Situational Vertigo Questionnaire (SVQ) [(29), adapted from Ref. (30)] is a 19-item questionnaire specifically aimed at identifying the presence of visual vertigo, a condition attributable to a defective vestibular compensation strategy, which is too dependent on the available visual information. “Visual vertigo” (31) is reported by patients with balance disorders in which symptoms are provoked or aggravated by specific disorienting visual context such as supermarkets (30), driving (32), movement of objects, or of the visual scene (33), which provoke abnormally large perceptual and postural responses. Visual vertigo is considered to be due to increased visual dependence with difficulty in resolving conflicts between visual and vestibule–proprioceptive inputs. The SVQ asks the patients to rate how much vertigo symptoms are provoked or exacerbated in environments with visual–vestibular conflict, or intense visual motion, and yields a score for each item between 0 (not at all) to 4 (very much); a “never experienced” answer can be given if the patient has never experienced the described situation. The total score will be then calculated as the sum of single item scores divided per 19 minus the number of never experienced situations (total score/19-number of “never experienced” answer).

The three questionnaires were translated into Italian and given to patients with acute or chronic dizziness for peripheral or central vestibular disorders. Internal consistency and reliability were analyzed for the translated questionnaires and their subscales. We then evaluated the correlation between the questionnaires’ scores and, for each questionnaire, the effects of demographical and clinical variables.

## Materials and Methods

### Translation and Cross-Cultural Adaptation

The final version of the DHI-I, ABC-I and SVQ-I can be found in the Supplementary Material for this article. We used the international guidelines for self-reported measures published by the American Association of Orthopedic Surgeons Outcome Committee (34) for the translation and cross-cultural adaptation process. Each questionnaire was translated into Italian by two independent bilingual translators. During a meeting, the two translators and Silvia Colnaghi synthesized the results of the Italian translations comparing them with the original version. This was followed by a back-translation into English by two independent bilingual persons who had no knowledge of the original versions. The pre-final questionnaires were produced by an expert committee consisting of the four translators, Silvia Colnaghi, and Maurizio Versino, taking into account all translations, written reports, and the original versions. The pre-final questionnaires were tested by Silvia Colnaghi and Maurizio Versino by interviewing 20 healthy subjects while they filled out the questionnaires. The objectives of the cognitive debriefing were to assess the comprehensibility of the pre-final versions. The transcripts of the patient interviews were analyzed by Silvia Colnaghi, who wrote the final version of the DHI-I, ABC-I, and SVQ-I.

The translations into Italian and the back translations of the original DHI, ABC scale, and SVQ were accomplished without major difficulties. The Italian versions were re-evaluated after back-translation procedure and the final version was produced after a test administration to 20 healthy Italian subjects, in order to evaluate the presence of dubious terms and sentences that can be difficult to understand. We also produced an assessment sheet to be filled in by the medical doctor and associated with the questionnaires. All participants answered the questions of the pre-final questionnaires spontaneously complying with expectations. Because of this, the comprehensibility of the DHI-I, ABC-I, and SVQ-I could be qualified as good.

### Study Population

This study was carried out in accordance with the recommendations of the institutional and national research committee with written informed consent from all subjects. All subjects gave written informed consent in accordance with the Declaration of Helsinki.

Patients were recruited from the National Neurological Institute C. Mondino Foundation and the ENT Clinic of Policlinico San Matteo in Pavia, and the ENT Department of the University of Siena. All patients who were referred to the outpatients vertigo services were asked to participate in this study. If a patient agreed, fulfilled the inclusion/exclusion criteria, and gave the written consent, he/she was included in the study. The research protocol received approval from the Ethics Committee of the National Neurological Institute C. Mondino and all the procedures were conducted in accordance with the Declaration of Helsinki. Informed consent was obtained from all individual participants included in the study.

Eligible patients needed to suffer from vertigo, dizziness, or unsteadiness associated with a central or peripheral vestibular disorder; further inclusion criteria were the following: age between 18 and 75 years, the ability to walk and to independently manage approximately 50% of daily tasks, and the ability to understand and speak Italian. Exclusion criteria were dizziness or unsteadiness exclusively due to cardiopulmonary diseases or musculoskeletal problems, severe paresis, spasticity, cerebellar ataxia, extrapyramidal diseases, or sensory ataxia, diagnosed dementia, psychiatric disorders, or blindness.

### Data Collection

For all patients, we collected demographic and clinical data and performed a standard neuro-otological examination including checking for the presence and the features of nystagmus (direction, amplitude, conjugacy and waveform, effect of preventing visual fixation), positional testing, hyperventilation, head shaking test, head impulse test, and Valsalva maneuver. Instrumental evaluation included caloric and rotational testing and instrumental head impulse testing (35–38). Clinical variables relevant for subsequent test of their association with the questionnaires’ scores were: disease duration, migraine diagnosis; visual vertigo; vestibular deficit (defined by clinical and instrumental evaluation); diagnosis (defined on the basis of anamnestic, clinical, and instrumental evaluation).

The three questionnaires were given to the patients in random order and answered by themselves (not by the caregivers). Thirty consecutive patients, who were assumed to have a stable health condition for the next 2 weeks, were asked to fill out the questionnaire twice, before and after a 2-week wash out period.

### Statistical Analysis

A statistician, Cristiana Rezzani, analyzed the data and performed the validation process, starting from descriptive statistics of the participants’ characteristics. The study of dimensionality was based on Exploratory Factor Analysis using the Principal Component Factors (PCF) method. The number of factors to be retained was evaluated on the basis of eigenvalues of the covariance matrix, according to Kaiser’s and Cattel’s criteria. According to Kaiser’s criteria, each factor with an eigenvalue greater than 1 should be retained. Cattel’s criteria are based on the interpretation of Cattel’s scree plot (39), the eigenvalues lying on the ordinate axis and the factor numbers on the abscissa axis; if the questionnaire is unidimensional, the first eigenvalue will be higher than the others, which will be well fitted by a single straight line. If the proportion of variability explained by the first dimension is greater than 50%, the questionnaire is unidimensional.

To evaluate the reliability of the questionnaires, we computed the Cronbach’s alpha (40), which is used as a reliability coefficient based on internal consistency. A high Cronbach’s alpha indicates high homogeneity of items; we considered 0.70 as the threshold above which the questionnaire can be considered reliable. In order to evaluate item homogeneity, we also computed the inter-item correlation matrix, corrected item-total correlation, and computed the “Cronbach’s alpha if item is deleted” value.

Test–retest reliability was evaluated by calculating the intraclass correlation coefficient (ICC) (41).

Correlations with demographical variables (age and disease duration) was evaluated by means of Spearman’s rank correlation coefficient, and association with clinical variables (vestibular signs, diagnosis, visual vertigo, migraine) by means of Kruskal–Wallis test. A *p*-value <0.05 was considered statistically significant.

## Results

**Figure 1:**
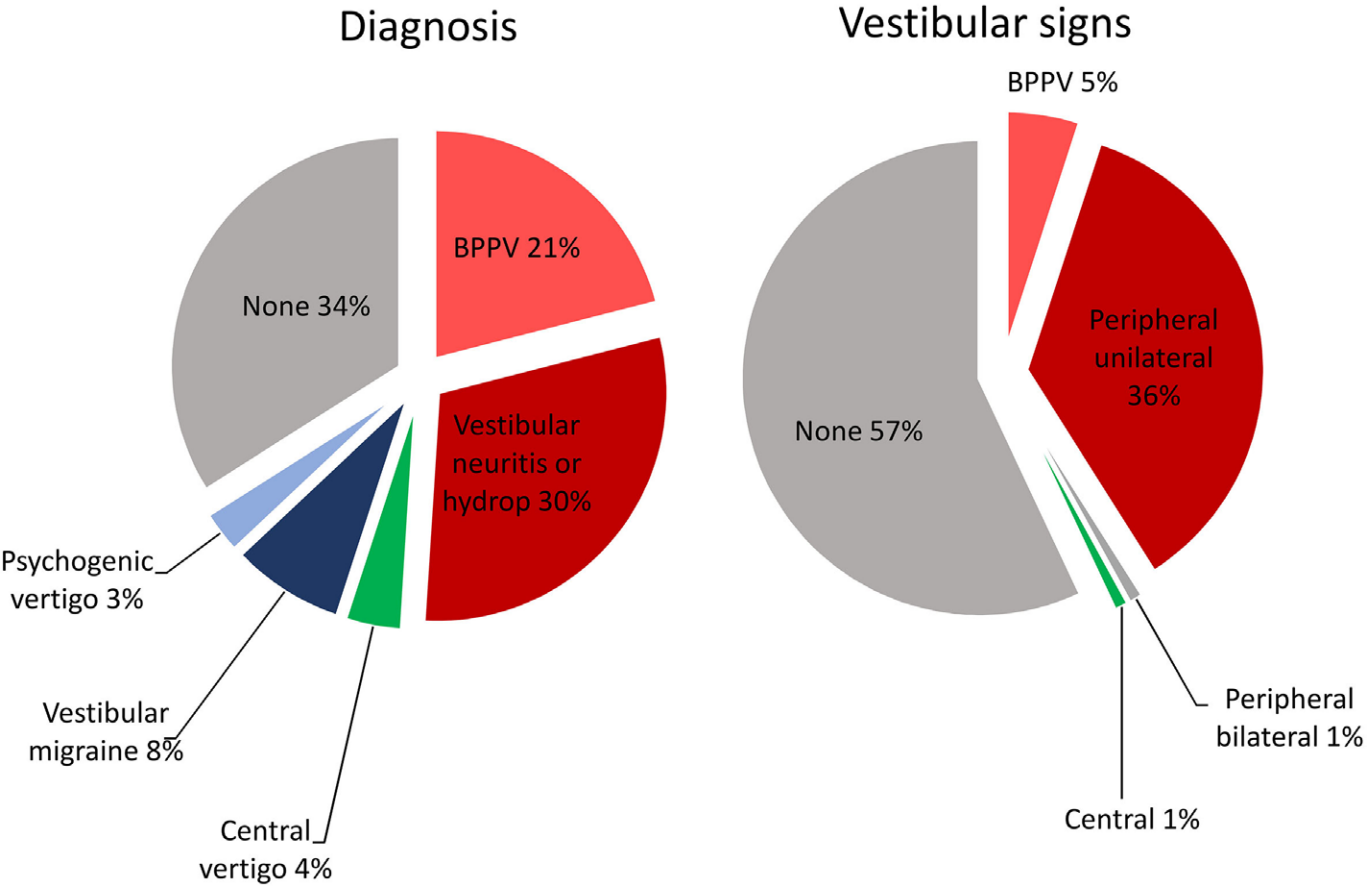
Vestibular diagnosis and signs distribution in the studied population. Diagnosis (left panel) was defined on the basis of anamnestic, clinical, and instrumental evaluation and included: benign paroxysmal positional vertigo (BPPV) (current or previous), vestibular neuritis, endolymphatic hydrop, central vertigo, vestibular migraine, and psychogenic vertigo. Vestibular signs (right panel) were evaluated by means of a standard neuro-otological examination and instrumental evaluation and classified as signs of BPPV, unilateral vestibular deficit (peripheral), brainstem or cerebellar dysfunction (central), and bilateral vestibular deficit (bilateral).

Study population included 316 patients (124 males, 192 females; mean age 53.5 years, SD 15.6 years); mean symptoms duration was 1.6 years (SD 3 years.). Vestibular signs and diagnosis were distributed as reported in Figure 1. Visual vertigo was identified in 0.2%, and migraine in 47.8% of the patients, but vestibular migraine was diagnosed only in 8% of the patients as the cause of dizziness and vestibular signs. Test–retest reliability group was composed by 17 males, 13 females; mean age 45.8 years, SD 18.5 years; mean symptom duration was 2 years (SD 4.5 years). Visual vertigo was identified in 0%, and migraine in 50% of these patients’ group. All patients completely and correctly filled out the questionnaires.

### Reliability of the Italian Version of the DHI, the Activity-Specific Balance Confidence Scale, and the SVQ

#### Dizziness Handicap Inventory

In order to test the unidimensionality of the physical, functional, and emotional subscales, we ran a series of exploratory factor analyses using the PCF method. The first component of Physical, Emotional, and Functional subscale explained 43, 40, and 39% of total variability, respectively. Only the first eigenvalue was above the threshold according to Kaiser’s criterion for the Physical subscale, while the first two were over one for the Emotional and Functional subscale. The graphic representation of the eigenvalues suggested the presence of a single latent dimension; indeed, in both cases, the second factors’ eigenvalues were slightly above 1.

The Cronbach’s alpha of the 7-item Physical subscale was 0.769. The covariance matrix showed no negative correlations (range 0.160–0.510), and the Cronbach’s alpha never increased if any item was deleted.

The Cronbach’s alpha of the 9-item Emotional subscale was 0.80. The covariance matrix range was 0.031–0.583, and the Cronbach’s alpha increased only minimally if either item 18, 20, or 22 was deleted.

The Cronbach’s alpha of the 9-item Functional subscale was 0.775. The covariance matrix range was 0.134–0.640. Cronbach’s alpha would increase if item 5 or 7 was deleted.

#### Activity-Specific Balance Confidence Scale

The questionnaire included 16 items showing two eigenvalues greater than 1, but the first dimension is responsible of 64% of total variance indicating a one-dimension tool. Cronbach’s alpha showed great reliability (alpha = 0.954). Inter-item correlation matrix range was 0.438–0.800, with no negative correlations. Corrected correlation and squared correlation values were very high.

#### Situational Vertigo Questionnaire

The questionnaire included 19 items showing two eigenvalues greater than 1, but the first dimension is responsible for 56% of total variance indicating a one-dimension tool. The SVQ also showed great reliability with alpha = 0.954. Inter-item correlation matrix range is 0.274–0.854 and both corrected correlation and squared correlation values are very high.

#### Test–Retest Reliability

**Figure 2:**
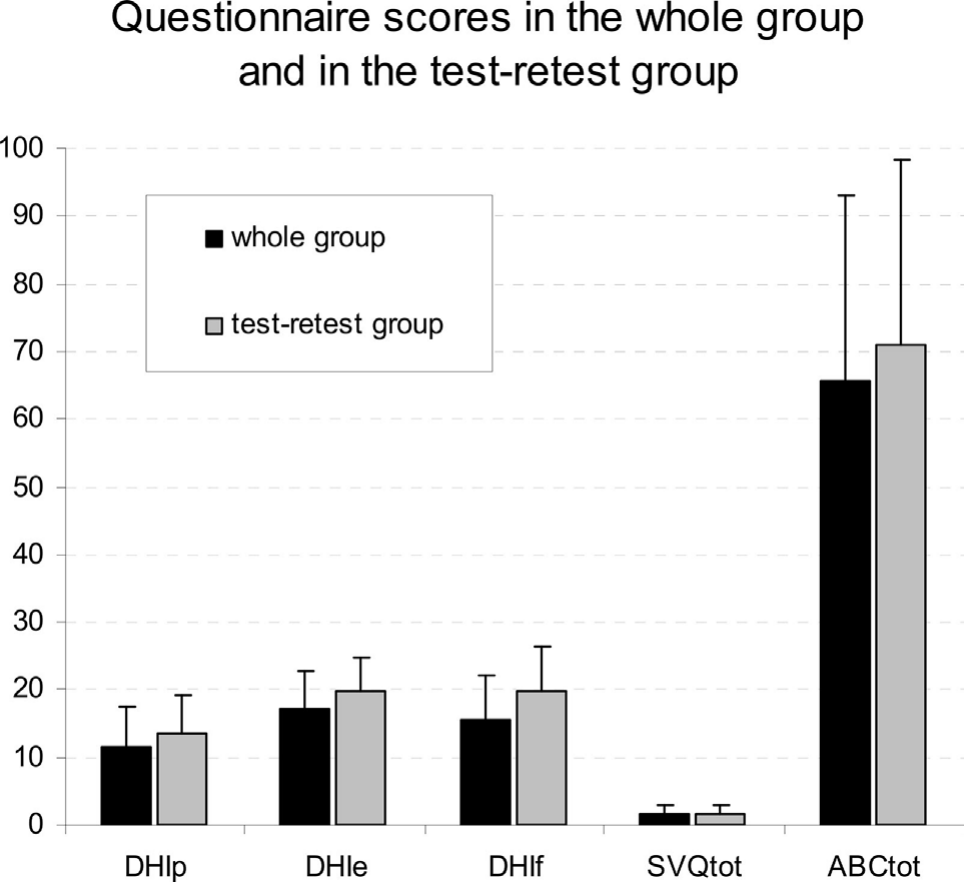
Distribution of scores of all patients and of the subgroup of 30 patients for the test–retest study. Mean and SD scores of the dizziness handicap inventory physical (DHI-p), emotional (DHI-e), and functional (DHI-f) subscales, in the situational vertigo questionnaire (SVQ) and in the activities balance confidence scale (ABC) in the whole patients group (black columns) and in subjects that answer the questionnaires twice for test–retest reliability evaluation (gray columns).

Figure 2 shows the distribution of scores of all patients and of the subgroup of 30 patients for the test–retest study.

Test–retest reliability was evaluated on a subgroup of 30 patients, in which the ICC was excellent for all the questionnaires [dizziness handicap inventory physical (DHI-p) ICC = 0.869; DHI-e ICC = 0.706; DHI-f ICC = 0.821; SVQ ICC = 0.949; ABS ICC = 0.978].

### Correlations Analysis and Effects of the Clinical Variables

Spearman’s rank correlation coefficients between the three questionnaires were all significant (Table 1) showing that the scales measure associated constructs or different aspects of the same construct.

**Table 1:** Spearman rank correlation coefficients (rho) between the three questionnaires.

		DHI-p	DHI-e	DHI-f	SVQ	ABC
DHI-p	Rho	1.000	0.285	0.464	−0.266	0.320
	*p*	–	**<0.001**	**<0.001**	**<0.001**	**<0.001**

DHI-e	Rho	0.285	1.000	0.474	−0.141	0.242
	*p*	**<0.001**	–	**<0.001**	**0.012**	**<0.001**

DHI-f	Rho	0.464	0.474	1.000	−0.149	0.353
	*p*	**<0.001**	**<0.001**	–	**0.008**	**<0.001**

SVQ	Rho	−0.266	−0.141	−0.149	1.000	−0.493
	*p*	**<0.001**	**0.012**	**0.008**	–	**<0.001**

ABC	Rho	0.320	0.242	0.353	−0.493	1.000
	*p*	**<0.001**	**<0.001**	**<0.001**	**<0.001**	–

*Correlations between the dizziness handicap inventory physical (DHI-p), emotional (DHI-e) and functional (DHI-f) subscales, the situational vertigo questionnaire (SVQ), and the activities balance confidence scale (ABC). Spearman rank correlation coefficient (rho) and *p*-values (two-tailed tests)*.

*Bold font is used for statistically significant correlations*.

Associations of the considered clinical variables on the questionnaires’ scores are shown in Table 2. All the scores significantly increased with age, with the exception of the SVQ. Disease duration positively correlated with the DHI-p, DHI-e, and DHI-f, but not with the SVQ and ABS score. Vestibular signs significantly correlated with the DHI-p (higher rank = 200.25, for brainstem or cerebellar signs), DHI-e (higher rank = 211.13, for bilateral vestibular signs), and DHI-f (higher rank = 165.94, for unilateral vestibular signs), but not with the SVQ and ABS score. Diagnosis only correlated with the DHI-p (higher rank = 180.10, for vestibular neuritis or endolymphatic hydrop) and DHI-e (higher rank = 170.10, for vestibular neuritis or endolymphatic hydrop) score. The presence of visual vertigo only correlated with the DHI-e score. The presence of migraine was the only variable correlating with the SVQ score.

**Table 2:** Effects of the clinical and demographical variables.

Clinical variables’ effects							
		Age	Disease duration	Vestibular signs	Diagnosis	Visual vertigo	Migraine
DHI-p	Rho/chi square	−0.155	0.134	11.983	17.167	0.027	0.118
	*p*	**0.007**	**0.022**	**0.017**	**0.009**	0.871	0.731

DHI-e	rho/chi square	−0.171	0.066	12.871	12.554	3.769	0.247
	*p*	**0.003**	0.257	**0.012**	**0.051**	**0.052**	0.619

DHI-f	Rho/chi square	−0.285	0.143	10.579	7.743	0.349	0.798
	*p*	**<0.001**	**0.014**	**0.032**	0.258	0.555	0.372

SVQ	Rho/chi square	0.002	0.029	1.969	5.022	0.536	4.240
	*p*	0.970	0.625	0.741	0.541	0.464	**0.039**

ABC	Rho/chi square	−0.197	−0.018	3.674	9.514	0.361	0.008
	*p*	**0.003**	0.797	0.452	0.147	0.548	0.930

*Effects of the demographical and clinical variables on the scores of the dizziness handicap inventory physical (DHI-p), emotional (DHI-e), and functional (DHI-f) subscales, the situational vertigo questionnaire (SVQ) and the activities balance confidence scale (ABC). Spearman’s rank correlation coefficient (rho), chi square from Kruskal–Wallis test, and *p-*values (two-tailed tests). Bold font is used for statistically significant effects*.

## Discussion

The ease of use and reliability of the three questionnaires were confirmed. Given that Cronbach’s alpha, a widely used measure of reliability, is considered good when values are above 0.70, the value obtained by the DHI, the ABC, and the SVQ emerges as an excellent result. The previous paper (22), which dealt with the validation of a DHI Italian version showed even higher Cronbach’s alpha scores as compared to ours. However, that paper considered a smaller number of patients, all of whom were in the acute phase of a peripheral vestibular disorder. We considered our sample to better match the situation in which the questionnaires are likely to be useful: we included patients with central vestibular disorders, and this can explain a larger variability than that which can be expected in a single disease, and, more importantly, we considered patients with long-lasting symptoms, namely, a condition in which the vestibular clinical and instrumental signs are not usually correlated to the subject’s self-feeling of imbalance.

Correlations between questionnaire scores and clinical data showed that age is associated with higher scores in all the DHI subscales and in the ABC scale, indicating that the physical, emotional, and functional impact of dizziness is greater in older patients and a relation between older age and the maximum perceived disability, which is known to be associated with an increased risk of falls (27). The SVQ score did not correlate with age, indicating that vertigo can be aggravated by specific visual context at any age. The SVQ score did not correlate with the diagnosis, included psychogenic vertigo and vestibular migraine and, surprisingly, the presence of symptoms of visual vertigo was not associated with a higher score at the SVQ, which is aimed at identifying the presence of this kind of symptoms, but this finding might be explained by the rare occurrence of these symptoms in the tested population (0.2%). On the other hand, the SVQ score was the only one that correlated with the presence of migraine as an associated symptom, suggesting that migraine patients can be more susceptible to experiencing difficulty in resolving visuo–vestibular conflicts. Increased visual dependence is a factor that can perpetuate symptoms in patients with balance disorders (31, 42, 43), and administration of the SVQ could help identify it and prompt specific rehabilitation treatment, incorporating desensitization exercises and simulator-based desensitization exposure (44).

Disease duration correlated with the physical and functional scale of the DHI, indicating how physical problems and inability build up with time. As expected, the presence of vestibular signs was associated with higher scores at the DHI-p and -f, with the higher ranking between DHI-p score and brainstem or cerebellar signs, indicating that such patients have a greater physical impairment, and between the DHI-f score and unilateral peripheral vestibular signs, which provokes, thereby, a greater restriction in everyday life. The DHI-e score also correlated with vestibular signs, particularly with bilateral peripheral signs, but these were detectable only in four patients of our population.

Finally, the diagnosis of vestibular neuritis or endolymphatic hydrops was associated with higher scores at the DHI-p and -e, indicating a relevant physical and emotional impact of these diseases, characterized by violent spells of vertigo that, in the case of hydrops, can recur.

The correlation of the DHI-e subscale score with the diagnosis of endolymphatic hydrops is in accordance with the association of Ménière disease with anxiety and depression, as shown by a previous study (45).

A possible limitation of the validation process in our study is that of the administration of three questionnaires at the same time. For instance, answering the first questionnaires could affect the answers to that following, and this effect could, in turn, amplify the correlations between questionnaire scores, or hide their natural variability. Furthermore, the great number of items could lead subjects to answer inaccurately, particularly to the last ones. To avoid these potential limitations, we administered the three questionnaires in random order and we found that correlations with clinical and demographical variables differed in the three questionnaires, suggesting that they measured different aspects of dizziness, despite having been administered in the same session.

In conclusion, we provided the Italian version of three self-administered questionnaires that aim at evaluating the functional limitations caused by the vestibular disorder and reliably assess the different aspects of dizziness, i.e., psychological, physical, and functional, as well as the risk of falling. These measures are specific to vestibular or balance disorder and can be used to evaluate quality of life, disability, and outcomes of intervention.

## Ethics Statement

This study was carried out in accordance with the recommendations of the local ethic committee with written informed consent from all subjects. All subjects gave written informed consent in accordance with the Declaration of Helsinki. The protocol was approved by the Ethic Committee of the National Neurological Institute C. Mondino Foundation.

## Author Contributions

SC, MV, and CR gave substantial contributions to the conception, design and interpretation of data, and drafted the work. MG and MCM gave substantial contributions to the interpretation of data and revised the work critically for important intellectual content. MMF, SQ, DN, and MMD gave substantial contributions to the acquisition of data and revised the work critically for important intellectual content. All the authors gave final approval of the version to be published and agreed to be accountable for all aspects of the work in ensuring that questions related to the accuracy or integrity of any part of the work are appropriately investigated and resolved.

## Conflict of Interest Statement

The authors declare that the research was conducted in the absence of any commercial or financial relationships that could be construed as a potential conflict of interest.
