# Supplementary material for: Validation of the Italian Version of the Dizziness Handicap Inventory, the Situational Vertigo Questionnaire, and the Activity-Specific Balance Confidence Scale for Peripheral and Central Vestibular Symptoms
Source: Front Neurol. 2017 Oct 10;8:528. doi: 10.3389/fneur.2017.00528 (PMC5641311; doi:10.3389/fneur.2017.00528)
Supplement: Supplementary file 1 [file data_sheet_1.pdf]

Dizziness Handicap Inventory (DHI-I)

RISPONDA ALLE DOMANDE IN BASE A COME È STATA LA SUA VERTIGINE NEGLI ULTIMI TEMPI ***(NON IN BASE A COME SI SENTE IN QUESTO PRECISO MOMENTO)***

P1.	I suoi disturbi peggiorano se guarda verso l’alto?	**Si**	**No**	**Qualche volta**
E2.	A causa dei suoi disturbi si sente frustrato?	**Si**	**No**	**Qualche volta**
F3.	A causa dei suoi disturbi limita le occasioni di viaggio per lavoro o per svago?	**Si**	**No**	**Qualche volta**
P4.	Camminare lungo il corridoio di un supermercato peggiora i suoi disturbi?	**Si**	**No**	**Qualche volta**
F5.	A causa dei suoi disturbi ha difficoltà a sdraiarsi nel letto o ad alzarsi dal letto?	**Si**	**No**	**Qualche volta**
F6.	I suoi disturbi riducono significativamente la sua partecipazione in occasioni sociali come uscire a cena, andare al cinema, a ballare o a delle feste?	**Si**	**No**	**Qualche volta**
F7.	A causa dei suoi disturbi ha difficoltà a leggere?	**Si**	**No**	**Qualche volta**
P8.	Praticare attività impegnative come uno sport, ballare, fare le pulizie o sparecchiare peggiora i suoi disturbi?	**Si**	**No**	**Qualche volta**
E9.	A causa dei suoi disturbi ha paura ad uscire di casa senza che qualcuno la accompagni?	**Si**	**No**	**Qualche volta**
E10.	A causa dei suoi disturbi si sente in imbarazzo di fronte ad estranei?	**Si**	**No**	**Qualche volta**
P11.	I movimenti rapidi della testa peggiorano i suoi disturbi?	**Si**	**No**	**Qualche volta**
F12.	A causa dei suoi disturbi evita i luoghi elevati?	**Si**	**No**	**Qualche volta**
P13.	Girarsi nel letto peggiora i suoi disturbi?	**Si**	**No**	**Qualche volta**
F14.	A causa dei suoi disturbi ha difficoltà a svolgere I lavori di casa più pesanti o I lavori in giardino?	**Si**	**No**	**Qualche volta**
E15.	A causa dei suoi disturbi teme che la gente possa pensare che sia drogato o ubriaco?	**Si**	**No**	**Qualche volta**
F16.	A causa dei suoi disturbi è difficile per lei camminare da solo?	**Si**	**No**	**Qualche volta**
P17.	Camminare lungo un marciapiede peggiora i suoi disturbi?	**Si**	**No**	**Qualche volta**
E18.	A causa dei suoi disturbi fa fatica a concentrarsi?	**Si**	**No**	**Qualche volta**
F19.	A causa dei suoi disturbi ha difficoltà a camminare in casa al buio?	**Si**	**No**	**Qualche volta**
E20.	A causa dei suoi disturbi ha paura di rimanere in casa da solo?	**Si**	**No**	**Qualche volta**
E21.	A causa dei suoi disturbi si sente inabilitato?	**Si**	**No**	**Qualche volta**
E22.	I suoi disturbi hanno creato tensione nelle sue relazioni con i familiari o con gli amici?	**Si**	**No**	**Qualche volta**
E23.	A causa dei suoi disturbi si sente depresso?	**Si**	**No**	**Qualche volta**
F24.	I suoi disturbi interferiscono con le sue responsabilità lavorative o domestiche?	**Si**	**No**	**Qualche volta**
P25.	Piegarsi in avanti peggiora i suoi disturbi?	**Si**	**No**	**Qualche volta**

Situtaional Vertigo Questionnaire (SVQ-I)

<u>Segni con una crocetta</u> quanto le condizioni descritte possono provocare o peggiorare la sua vertigine

0 = Per niente | **1 =** Molto poco | **2 =** Un poco | **3 =** Abbastanza | **4 =** Molto

Se non si è mai trovato nella condizione descritta segni: **Non l'ho mai fatto**

In macchina come passeggero percorrendo una strada dritta e piatta	**0**	**1**	**2**	**3**	**4**	**Non l'ho mai fatto**
In macchina come passeggero percorrendo una strada curva o sterrata	**0**	**1**	**2**	**3**	**4**	**Non l'ho mai fatto**
Camminando lungo la corsia di un supermercato	**0**	**1**	**2**	**3**	**4**	**Non l'ho mai fatto**
In piedi in un ascensore quando l'ascensore si ferma	**0**	**1**	**2**	**3**	**4**	**Non l'ho mai fatto**
In piedi in un ascensore mentre si muove ad una velocità costante	**0**	**1**	**2**	**3**	**4**	**Non l'ho mai fatto**
In automobile a velocità costante	**0**	**1**	**2**	**3**	**4**	**Non l'ho mai fatto**
In automobile alla partenza o frenando	**0**	**1**	**2**	**3**	**4**	**Non l'ho mai fatto**
Stando in piedi nel mezzo di un grande spazio aperto (ad esempio un parco o una piazza)	**0**	**1**	**2**	**3**	**4**	**Non l'ho mai fatto**
Seduto su un autobus	**0**	**1**	**2**	**3**	**4**	**Non l'ho mai fatto**
In piedi su un autobus	**0**	**1**	**2**	**3**	**4**	**Non l'ho mai fatto**
In un luogo elevato	**0**	**1**	**2**	**3**	**4**	**Non l'ho mai fatto**
Guardando in televisione o al cinema delle scene in movimento	**0**	**1**	**2**	**3**	**4**	**Non l'ho mai fatto**
Utilizzando le scale mobili	**0**	**1**	**2**	**3**	**4**	**Non l'ho mai fatto**
Guardando superfici rigate o in movimento (ad esempio delle tende colorate, le veneziane, l'acqua che scorre)	**0**	**1**	**2**	**3**	**4**	**Non l'ho mai fatto**
Guardando scorrere lo schermo del computer	**0**	**1**	**2**	**3**	**4**	**Non l'ho mai fatto**
Attraversando una galleria e guardando le luci su un lato	**0**	**1**	**2**	**3**	**4**	**Non l'ho mai fatto**
Attraversando una galleria e guardando le luci alla fine della galleria stessa	**0**	**1**	**2**	**3**	**4**	**Non l'ho mai fatto**
Guidando un'automobile sulla salita di una collina, lungo una curva o in spazi ampi	**0**	**1**	**2**	**3**	**4**	**Non l'ho mai fatto**
Guardando il flusso del traffico o un treno in movimento (ad esempio cercando di attraversare la strada o alla stazione)	**0**	**1**	**2**	**3**	**4**	**Non l'ho mai fatto**

Activity specific Balance Confidence scale (ABCs-I)

Risponda alle domande con un numero da 0 a 100 che significa:
0 = minima sicurezza
100 = massima sicurezza

“quanto si sente sicuro di mantenere l’equilibrio se…

…cammina dentro casa?	____
…sale o scende le scale?	____
… si china per raccogliere una ciabatta dal ripiano più basso dell’armadio?	____
… si allunga per prendere una lattina da un ripiano all’altezza dei suoi occhi?	____
… si alza sulle punte per raggiungere qualcosa sopra alla sua testa?	____
… si allunga per prendere qualcosa mentre è seduto?	____
…pulisce il pavimento?	____
… cammina da casa fino alla macchina parcheggiata sul viale?	____
…sale o scende dalla macchina?	____
… cammina attraverso il parcheggio fino al supermercato?	____
…sale o scende una rampa?	____
… cammina in un supermercato pieno di gente che le passa accanto velocemente?	____
… viene spinto dalle persone mentre attraversa il supermercato?	____
… sale o scende da una scala mobile mentre si tiene al corrimano?	____
… sale o scende da una scala mobile mentre regge dei pacchi, non potendo tenersi al corrimano?	____
…cammina su un marciapiede ghiacciato?	____
